# Immune challenge reduces daily activity period in free-living birds for three weeks

**DOI:** 10.1098/rspb.2023.0794

**Published:** 2023-08-30

**Authors:** Rosie J. Lennon, Shivani Ronanki, Arne Hegemann

**Affiliations:** Department of Biology, Lund University, Ecology Building, 223 62 Lund, Sweden

**Keywords:** eco-physiology, eco-immunology, inflammation, immunity, life-history ecology, pathogen

## Abstract

Non-lethal infections are common in free-living animals and the associated sickness behaviours can impact crucial life-history trade-offs. However, little is known about the duration and extent of such sickness behaviours in free-living animals, and consequently how they affect life-history decisions. Here, free-living Eurasian blackbirds, *Turdus merula*, were immune-challenged with lipopolysaccharide (LPS) to mimic a bacterial infection and their behaviour was monitored for up to 48 days using accelerometers. As expected, immune-challenged birds were less active than controls within the first 24 h. Unexpectedly, this reduced activity remained detectable for 20 days, before both groups returned to similar activity levels. Furthermore, activity was positively correlated with a pre-experimental index of complement activity, but only in immune-challenged birds, suggesting that sickness behaviours are modulated by constitutive immune function. Differences in daily activity levels stemmed from immune-challenged birds resting earlier at dusk than control birds, while activity levels between groups were similar during core daytime hours. Overall, activity was reduced by 19% in immune-challenged birds and they were on average almost 1 h less active per day for 20 days. This unexpected longevity in sickness behaviour may have severe implications during energy-intense annual-cycle stages (e.g. breeding, migration, winter). Thus, our data help to understand the consequences of non-lethal infections on free-living animals.

## 1. Introduction

A pathogenic challenge to the immune system can occur at any point during an animal's lifespan and affect the success of any ongoing life-cycle event. The capacity to deal with an immune challenge can therefore influence numerous life-history decisions. At the onset of infection, a suite of behavioural, physiological, immunological and metabolic mechanisms are activated, which are collectively called the acute phase response (APR) [1]. Metabolic requirements during an immune response (inclusive of the APR) can increase by 5–15%, which is significant for free-living animals that may experience energetic constraints [2]. To manage the metabolic cost and other associated adverse effects of an infection, individuals may undergo a suite of physiological and behavioural adjustments that are dependent on internal (e.g. individual physiology) and external factors (e.g. environmental resources and cues). As such, individuals experiencing an immune challenge, and thus modulating the APR, can suffer behavioural disruptions to both day-to-day (e.g. foraging effort, sexual signalling and predator avoidance) and key life events (e.g. breeding investment and migration), all of which can ultimately affect fitness [3–11]. Therefore, understanding the behavioural implications of immune-function-induced trade-offs during an infection is important for deciphering how host–pathogen interactions may influence species ecology and evolution.

‘Sickness behaviour’ is a term used to describe a behavioural alteration triggered by the APR that can also energetically recoup what is invested into physiological processes of the immune response [12,13]. Key sickness behaviours include reduced feeding and drinking, increased somnolence (also known as sleepiness or drowsiness), alterations to body temperature, and changes to social and grooming behaviours, which collectively manifest as an overall reduction in activity [8,14–18]. In particular, increased somnolence in birds is often reported within hours of APR onset, and in mammals immune challenges are known to increase the amount of slow-wave sleep [12,14]. Consequently, decreased alertness and activity levels induced by sickness behaviours also can increase predation risk in animals undergoing infection [9,19]. Previous studies have also reported that sickness behaviours are modulated depending on the availability of energy resources (e.g. seasonal) and/or fitness advantages associated with maintaining activity levels at the time of challenge [13,20–22]. Consequently, there is some consistency across multiple taxa of the types of sickness behaviours observed, but the longevity, pattern and variation of these behavioural alterations remain largely unquantified, and particularly so in free-living animals [23].

Sickness behaviours are mechanistically activated by the release of proinflammatory cytokines (IL-1β, IL-6, TNF-α) during the APR, which elicit various neurological pathways via the primary afferent nerves, the vagus nerve and blood–brain barrier [1,23]. The intensity and longevity of sickness behaviours may therefore depend on the parameters of this pathway, inclusive of an individual's baseline immunological capacity. In particular, high levels of constitutive (baseline) immune function may be advantageous during a pathogenic challenge ([24,25], but see [26]). Furthermore, several studies have shown links between constitutive immune function or an immune response upon an immune challenge and subsequent survival ([24,27–30], but see [31]). However, it is currently unclear if, and to what extent, constitutive immune function is predictive of sickness behaviours in free-living animals.

Hypotheses relating to sickness behaviours are commonly tested by administering an endotoxin that promotes an APR, thus mimicking the first stages of a bacterial infection without causing an actual microbe-invasive infection [32–34]. In birds, the majority of studies concerning sickness behaviours have been conducted in controlled aviary/cage-based experiments, or short-term field experiments; to our knowledge, the longest duration of any such experiment does not exceed 22 days [15] and in all instances behaviours are monitored at selected timepoints within the experimental time frame, rather than continuously. In aviary experiments, sickness behaviours (including reduced activity (feeding, drinking) and increased somnolence (resting)) resulting from mimicked immune challenges have mostly been measured within 48 h of treatment [8,13,17,20,35,36], with a couple of exceptions of extended studies recording effects up to 5 and 9 days post-challenge [21,37]. In a study on caged house finches (*Haemorhous mexicanus*) using a real bacterial pathogen (*Mycoplasma gallisepticum*), reduced activity (perching, eating, walking, flying) was observed in infected birds on day 6 but not on day 22 post-infection [15]. Comparatively fewer sickness behaviours have been examined in free-living birds; Adelman *et al.* [38] and Owen-Ashley *et al.* [8] observed reduced activity, territorial aggression and song in sparrow species within the first 24 h post-challenge. There is also evidence that naturally occurring immune challenges in wild birds and mammals can reduce regional movements, foraging activity and migration [7,18,39–41], but those studies are usually correlative and/or not done during peak infection. Taken together, there are a paucity of data to examine sickness behaviours in free-living animals from the onset of an immune challenge over an extended period of time, and most studies have only captured a snapshot of behaviour at either one or multiple timepoints during an infection, rather than consistently until any effect dissipates. As such, the plasticity and longevity of sickness behaviours in wild animals remain largely unknown, especially in terms of how these are managed as part of diurnal activity rhythms and how this could translate to associated fitness costs.

Here, a field-based design was used to continuously measure the behavioural response of free-living birds to an experimentally mimicked bacterial infection over a period of up to 48 days using state-of-the-art accelerometers. Specifically, the aims were to (1) assess whether overall levels of activity in birds changed in response to the immune challenge (e.g. level of activity during active hours), (2) analyse whether there were any differences in diurnal patterns of activity between immune-challenged and control birds (e.g. number of active versus inactive hours), and (3) investigate whether baseline immune function prior to the immune challenge is associated with how birds behaviourally responded to the treatment. It is expected that immune-challenged birds experiencing the APR will express sickness behaviours that will manifest in lower levels of activity compared with control birds that are not immune-challenged. Furthermore, patterns of activity in immune-challenged birds may be affected by elongated resting periods, as is a common sickness behaviour, but higher levels of baseline immune function may correlate with lower behavioural impacts of the immune challenge.

## 2. Methods

### (a) Data collection

Data were collected from an urban population of Eurasian blackbirds (*Turdus merula*) situated in Lund, Sweden (55.7047°N, 13.1910°E). Birds were first captured between 28 July and 4 October in 2019 or 2020 (electronic supplementary material, figure S1). The breeding season in this population ends in early August and 78% of birds included in this study had already started moulting at the start of the experiment. Those birds that had not started moulting at capture may still have been caring for nestlings or fledglings. Birds were recaptured in the subsequent year between May and September. Birds were caught using mist nets in either the morning or the evening. On average, birds were extracted from the net and approximately 300 μl of blood was collected from the jugular vein within 5 min of birds entering the net (range: 3–12 min). Body mass, tarsus length and wing length (maximum chord length) were measured to the nearest 0.1 g, 0.1 mm and 1 mm, respectively. Birds were aged and sexed based on plumage characteristics [42]. Accelerometers (designed and manufactured by the Technical Lab, Department of Biology, Lund University, Sweden) were fitted at the first capture event using an elastic cord leg loop harness [43]. In total, 45 accelerometers were deployed (2019: *n* = 20, 2020: *n* = 25; 27 males, 18 females). Accelerometers recorded the level of lateral movement five times per hour to produce an hourly activity score between zero (no movement) and 60 (constant, high-intensity movement; for details see [44]). Accelerometers were always fitted in conjunction with a radio transmitter (NanoTags, Lotek Wireless, UK), which were used as part of a larger study. The total combined weight of the accelerometer and radio tag was approximately 3.0 g (including the elastic cord), which is equivalent to 3% of the average body weight of the birds included in this study. Finally, each bird was assigned to either a control or experimental group, with males and females distributed evenly between the two (electronic supplementary material, figure S1). Birds belonging to the experimental group received an immune challenge to mimic a bacterial infection via a subcutaneous injection of bacterial lipopolysaccharide (LPS: an immunogenic molecule found in the membrane of Gram-negative bacteria; specifically LPS from *Escherichia coli* 055:B5; Sigma-Aldrich, Sweden; product number L2880) at a concentration of 1 µg LPS g^−1^ body mass, dissolved in 2 µl phosphate-buffered saline (PBS), which is a standard concentration used in many passerines [3,45,46]. Hence, each blackbird received an injection volume depending on its body mass (e.g. a 90 g blackbird received an injection volume of 180 µl). Control birds received no injection because puncturing the skin and injecting a vehicle alone (e.g. PBS) may also cause inflammation. Consequently, the experimental responses must be viewed as the result of both the LPS and the injection procedure. This combination of effectors does not pose interpretational problems for this study, since the central interest was to induce an innate (inflammatory) immune response and compare this with the absence of such a response, not the effects of LPS *per se* (see also [3,35,47]). Blood was stored on ice until being returned to the laboratory on the same day, where plasma and red blood cells were separated by centrifugation (6000 r.p.m. (222*g*), 10 min) and stored at −50°C until analysis.

### (b) Available activity data

Of the 45 accelerometers deployed, 23 were retrieved (10 from the 2019 cohort, 13 from the 2020 cohort); however, one tag failed to record any data. Of the remaining 22 accelerometers, 12 belonged to control birds (3 females, 9 males) and 10 to immune-challenged birds (5 females, 5 males; electronic supplementary material, table S1). Across both years, the earliest deployment date for a retrieved tag was 28 July and the latest deployment date was the 20 September (9/22 accelerometers had non-unique deployment dates: electronic supplementary material, figure S1). Among individuals, activity data were available for 24–310 days after the deployment date (average: 250 days). Within the experimental group, one individual displayed activity scores that were approximately three times higher than the other birds in the first 20 days (and particularly in the first 24 h) after treatment (electronic supplementary material, figure S2). Since no methodological abnormalities were detected that would justify exclusion of this bird, this individual was kept in all analyses and as such the model outputs can be considered conservative.

**Figure 1.**
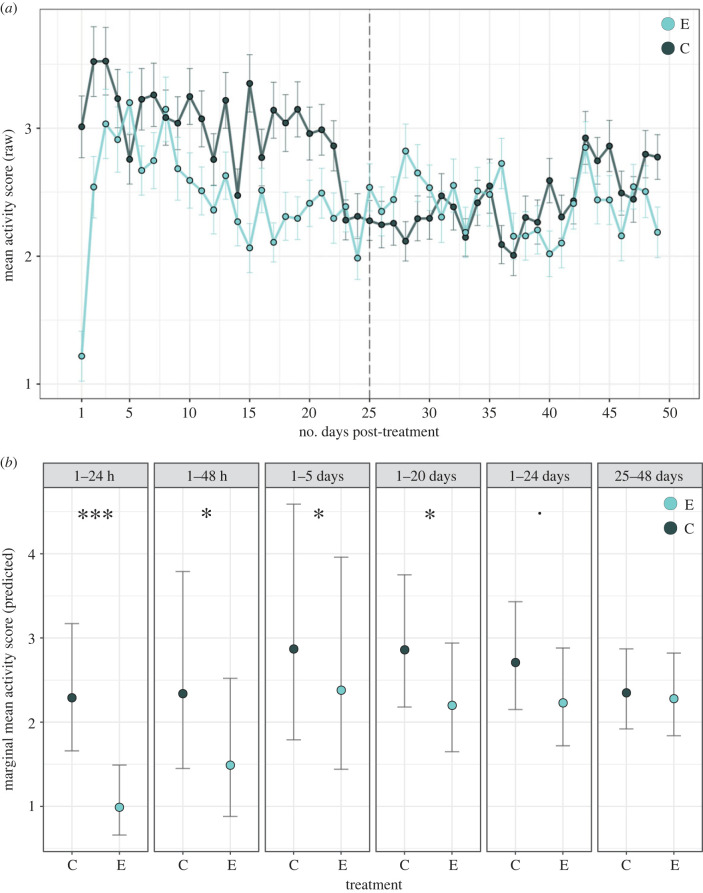
(*a*) Mean hourly activity scores of blackbirds for daytime hours (calculated from raw data), with standard error bars. Data displayed for control (C; birds that received no injection) and experimental (E; birds injected with LPS) groups during the first 48 days after treatment. The follow-up period (days 25–48) is to the right of the dashed line, whereas all other analysed time frames (up to 24 days) are to the left. (*b*) Predicted marginal means for the activity score of control and experimental blackbirds, calculated from generalized linear mixed models for each time frame analysed (see Methods). Error bars represent the lower and upper confidence intervals for the predicted mean (95%). Asterisks denote significant effect estimates for activity score between the two treatment groups with ***p < 0.001; *p < 0.05; ^**.**^*p* < 0.1.

Hourly activity scores were extracted from each accelerometer and checked for mechanical time lags and/or restarts over the course of deployment. The data were trimmed so that the first activity score represented at least the first full 50 min after the bird was released (e.g. if the bird was released more than 10 min past the hour, the data series started at the next full hour). Data for all birds (*n* = 22) were obtained for the first 24 days post-treatment (i.e. days 1–24), which was the least number of days any one tag recorded data for. This time frame was further split into four time windows (*n* = 22 individuals): 1–24 h, 1–48 h, 1–5 days, 1–20 days, 1–24 days post-treatment. Analysing the data using these additive time windows allowed the longevity of any behavioural changes to be identified, rather than examining concise time frames (though an alternative approach is presented in electronic supplementary material, note S1, figure S3). The first three time periods correspond to known durations of either behavioural or physiological effects of an LPS challenge in birds under aviary conditions [8,37], while the 20-day period was chosen after visual assessment of the activity data over the full time series (figure 1*a*). Additionally, days 25–48 post-treatment (*n* = 21 individuals) were analysed as a follow-up time period to ascertain the extent of any effects observed. All 21 accelerometers had collected data up to and including day 48. ‘Day’ is defined here as each consecutive 24 h period after the bird received either the experimental or control treatment (i.e. the release time of each bird).

### (c) Immune function assays

Plasma samples were used to quantify four indices of baseline (constitutive) innate immune function prior to the immune challenge, using three assays. Firstly, bacterial killing capacity was measured following the method described in [48], with modifications according to [49]. This assay measures an integrative ability of plasma to remove a pathogen (here *E. coli*) and has been shown to have predictive capacity for surviving a naturally occurring disease outbreak [24]. All plasma samples had been frozen for at least 60 days before assaying and hence bacterial killing capacity is expected to have reached its stable phase after initial loss of activity [50]. Based on a pilot study, the optimal concentrations for blackbirds were 3 µl plasma and 5 µl 3.2^8^ *E. coli* solution. All samples were run in triplicate and the resulting bacterial growth was measured at 600 nm using a plate reader. Any outliers within each triplicate were identified and removed with a coefficient of variation threshold of 20%, and remaining values were averaged. Secondly, haptoglobin was measured via a commercial colometric kit (TP801; Tri-Delta Diagnostics, Maynooth, Ireland), following the same protocol as [25]. Haptoglobin is an acute-phase protein that is associated with the initial stages of an immune response [51] and baseline haptoglobin concentrations can predict changes in haptoglobin during an infection [25]. And finally, both lytic enzyme activity of the complement system (measured as lysis) and non-specific natural antibodies (measured as agglutination) were measured. In particular, lysis has been shown to have predictive capacity of survival in free-living birds [27,30]. The haemagglutination–haemolysis assay was used following [52] but 20 µl of plasma was used instead of 25 µl (rabbit blood source: Envigo, UK: Alsevers, S.BC-0009). Scans of individual samples were randomized among all plates and scored at least twice blindly with respect to individual ID and treatment group. If scores differed by >1, then a third score was taken and the median was taken as a final value. Based on a chicken control that was run in duplicate on each plate, the coefficient of variation for within-plate variation was 7 and 6%, whereas the between-plate variation was 17 and 11% for lysis and agglutination, respectively. Samples were defrosted and refrozen between each assay, but these assays are robust to repeated freeze–thaw cycles [53].

### (d) Statistical analyses

All statistical analyses were carried out in R (v. 4.1.1, [54]). Generalized linear mixed models (GLMMs) were used to analyse the data using the package ‘glmmTMB’ (v. 1.1.2.3, [55]) and model fit was assessed using DHARMa (v. 0.4.4, [56]).

#### (i) Overall levels of activity

To reduce zero-inflation within the data, all activity scores before dawn (defined as when morning nautical twilight ends and morning civil twilight starts) and after dusk (defined as when evening nautical twilight starts) were omitted using the ‘suncalc’ package (v. 0.5.0, [57]). The resultant hourly activity score data (daytime only) for each individual were used for GLMMs to analyse daytime activity. Individual models were run using the first 24 h, 48 h, 5 days, 20 days, 24 days and 25–48 days post-treatment. Model fit in all instances was assessed via visualization of simulated residuals and *QQ*-plots, and statistical tests for overdispersion, outliers and zero-inflation.

To assess activity during daytime hours, hourly activity scores were analysed for each individual as a function of treatment (control versus immune-challenged) as a fixed effect, and sex, ordinal day, year and time of capture (i.e. whether the bird was caught in the morning or the evening) as covariates. All covariates were included in the model to account for any natural variation, rather than being modelled explicitly to investigate how activity differed in relation to them. Each model also included an autoregressive covariance matrix using the ‘ar1()’ function in glmmTMB. In the 5-, 20-, 24- and 25–48-day models, the covariance matrix ordinal day was used, whereas the hourly models used capture hour. This was necessary owing to temporal autocorrelation within the activity score data and the fact that accelerometers were deployed on different dates within each year. The date of capture (ordinal day) in particular may influence daily activity patterns owing to differences in day length as well as the proximity of capture to the moulting season and progress of moulting, when birds generally become less active [58,59]. The interaction between treatment and capture time was included in all models because LPS responses can vary depending on the time of day that the challenge is administered [37]. Finally, individual ID was included as a random factor to control for repeated measurements. All models were run using a zero-inflated log link negative binomial distribution (quadratic or linear parameterization). Marginal means for activity score in relation to each treatment group were calculated using the ‘ggeffect’ function from the ‘ggeffects’ package [60]. Separate marginal means were extracted from each GLMM for each time frame analysed.

#### (ii) Diurnal patterns of activity

To analyse whether there were any alterations to the daily first and last hour of activity within control and immune-challenged birds (hereafter termed as wakefulness, even though we could not distinguish whether birds slept or just sat still), the full dataset (including night-time hours) was used. Specifically, the first hour and last hour when the activity score was >1 (e.g. birds with a score of 1–60 were active, compared with 0 being not active; maximum activity score recorded: 24) were extracted for each 24 h period (midnight to midnight). The difference between these times and dawn/dusk (rounded up to the nearest full hour) was then calculated for each day, for each individual bird, and the median difference was used for analysis to account for pseudo replication. To account for a mixture of positive and negative values and to acquire robust model outputs, non-parametric Mann–Whitney *U*-tests were used to test for any differences in wakefulness between birds in either treatment group. Five datapoints (from two individuals; total number of datapoints: 2097) were removed from this analysis where it was not possible to explicitly determine the first or last hour of activity (where birds were either active consistently through the night (*n* = 1) or active inconsistently for less than 6 h of the day (*n* = 4)).

To analyse ‘core’ daytime hours, any hours that constituted either the first or last hour of activity across all individuals were excluded from the dataset. To analyse the levels of activity within these core daytime hours (only), the same GLMM structure was used as for full daytime hours, except only treatment was included as a fixed effect and no covariance structure was included. This simpler model structure compared with the above models was necessary owing to issues with model convergence. Activity score for core hours was modelled using a log link negative binomial distribution (quadratic parameterization; a zero-inflation parameter was unnecessary). The same time periods were analysed as for the full daytime hours.

#### (iii) Baseline immune function and activity

To test if baseline (pre-challenge) immune function had predictive capacity of the sickness behaviour within the first 24 h, Kendall rank tau coefficient was used to assess whether there was any correlation between measures of baseline immune function and the mean activity score (excluding night-time hours) of control and immune-challenged birds during the first 24 h. In cases where there was a significant correlation, we also tested the subsequent time period (1–48 h), but adjusted the significance value by *p* = 0.05/2 to account for multiple testing. In case there was still an effect during this time period, we also tested for the period 1–5 days, with *p* = 0.05/3 as new significance threshold. Only birds challenged in the morning were included in this analysis to reduce the variation in immune function coming from diurnal patterns [61].

## 3. Results

### (a) Overall levels of activity

Activity was significantly reduced in immune-challenged birds within the first 24 h, 48 h, 5 days and 20 days post-treatment (table 1; figure 1). Mean activity in the experimental group was 59, 41, 19 and 19% lower than in the control group within the first 24 h, 48 h, 5 days and 20 days post-treatment, respectively (please note that the effect sizes for the longer periods are always an average for the entire period starting with the onset of the experiment). Independently of treatment, females were significantly more active than male birds in all models within the first 24 days post-challenge, and activity scores significantly decreased with ordinal day except for the first 48 h (table 1).

**Table 1.** Generalized linear mixed model outputs for the analysis of hourly activity scores during daytime hours in blackbirds. Output values for all fixed effects and interactions are displayed for all time periods analysed (e.g. number of hours or days after lipopolysaccharide injection or control treatment was administered), including the follow-up period of 25–48 days post-treatment (see Methods for model details). Significant effect estimates are shown in bold, and reference level for factorial fixed effects is shown in brackets. E: experimental group; pm: evening-caught birds.

model	treatment (E)			sex (M)			year (2020)			ordinal day			capture time (pm)			treatment: capture time		
	effect	s.e.	*p*	effect	s.e.	*p*	effect	s.e.	*p*	effect	s.e.	*p*	effect	s.e.	*p*	effect	s.e.	*p*
1–24 h	−1.133	0.301	**<0.001**	−0.811	0.260	**0.002**	−0.504	0.366	0.169	−0.029	0.012	**0.016**	0.296	0.434	0.494	0.749	0.526	0.154
1–48 h	−0.701	0.297	**0.018**	−0.776	0.261	**0.003**	−0.227	0.376	0.545	−0.018	0.013	0.175	0.403	0.448	0.368	0.536	0.533	0.314
1–5 days^a^	−0.472	0.220	**0.032**	−0.743	0.206	**<0.001**	−0.581	0.310	0.061	−0.025	0.013	**0.050**	0.287	0.326	0.378	0.776	0.413	0.060
1–20 days^a^	−0.348	0.149	**0.019**	−0.439	0.134	**0.001**	−0.336	0.169	**0.047**	−0.017	0.005	**<0.001**	−0.144	0.205	0.480	0.365	0.278	0.189
1–24 days^a^	−0.304	0.160	0.058	−0.389	0.144	**0.007**	−0.177	0.176	0.316	−0.013	0.003	**<0.001**	−0.112	0.219	0.609	0.368	0.299	0.219
25–48 days	−0.134	0.136	0.326	−0.078	0.121	0.520	0.013	0.148	0.928	<0.001	0.002	0.856	−0.438	0.184	**0.017**	0.331	0.251	0.186

^a^Model residuals significantly non-uniform—see electronic supplementary material, table S2.

When the follow-up time frame of days 25–48 was analysed, there was no significant difference in activity scores between control and immune-challenged birds (table 1, figure 1), strongly suggesting that the difference in activity scores during the first 20 days after the experimental start is indeed a consequence of the treatment and not a by-chance difference in behaviour between the groups.

Variance introduced by individual ID and the date of activity was approximately equal in all models, except for the 5-day (all daytime hours) model, which had higher variance values. See electronic supplementary material, tables S2 and S3 for all model outputs relating to random effects and model fit.

### (b) Diurnal patterns of activity

**Figure 2.**
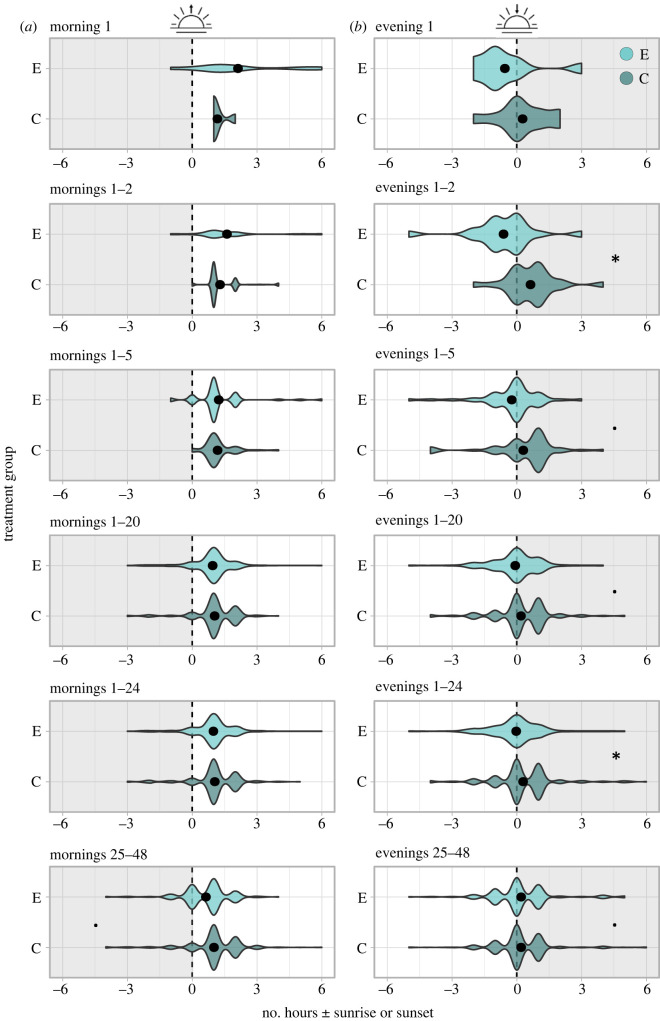
Distribution of the first hour of activity at (*a*) dawn or last hour of activity at (*b*) dusk. Data displayed for immune-challenged (E: birds injected with LPS) and control (C: birds that received no injection) blackbirds. Dawn/dusk are represented by dashed vertical lines; violin plots depict the density of the first or last hour of activity before (to the left of the dashed lines) or after (to the right of the dashed lines) dawn/dusk. Filled points are the median first or last hour of activity for either treatment group. Each panel represents a different time window after the experiment started: the first evening/morning, and the first 2, 5, 20 and 24 mornings/evenings; data were analysed for 22 birds for each time period. An asterisk indicates where the first/last hour of activity significantly differs between the two treatment groups, whereas a ‘^**.**^’ indicates if there was an observed trend (*p* > 0.05 and less than 0.1).

The last hour of activity before dusk was earlier in immune-challenged birds compared with control birds in the first 2, 5, 20 and 24 evenings post-treatment. This effect was significant for 2 and 24 days, and marginally non-significant for 5 and 20 days (table 2; figure 2). There was no significant difference in the first hour of activity at dawn between control and immune-challenged birds in any of the periods (1–24 h, 1–48 h, 1–5 days, 1–20 days, 1–24 days; table 2; figure 2). Collectively, the total number of hours that birds were active per day (i.e. activity score >1) was marginally higher in control birds (13.8 h day^−1^) than immune-challenged birds (13.1 h day^−1^) within the first 24 days post-treatment. Thus, control birds were approximately 0.7 h more active each day than immune-challenged birds (Mann–Whitney *U*: *W* = 74, *p* = 0.356).

**Table 2.** Analysis output to test whether the first/last hour of activity at dawn/dusk differs between immune-challenged and control blackbirds. Significant group differences are highlighted in bold. The direction of effect is detailed for significant correlations.

mornings/evenings post-treatment	Mann–Whitney		mean no. hours birds were first/last active at sunrise/sunset (s.e.)^a^	
	*W*	*p*	control	experimental
*dawn (mornings)*				
1	35.0	0.255	1.2 (0.11)	2.1 (0.81)
1–2	42.5	0.412	1.3 (0.15)	1.6 (0.38)
1–5	54.0	0.315	1.2 (0.09)	1.2 (0.18)
1–20	65.0	0.411	1.0 (0.07)	0.9 (0.07)
1–24	75.0	0.109	1.0 (0.06)	1.0 (0.07)
25–48	69.5	0.079	1.0 (0.07)	0.6 (0.07)
*dusk (evenings)*				
1	64.0	0.117	0.3 (0.36	−0.6 (0.50)
1–2	98.5	**0.011**	0.6 (0.25)	−0.6 (0.37)
1–5	85.0	0.075	0.3 (0.21)	−0.2 (0.20)
1–20	80.0	0.055	0.2 (0.09)	−0.1 (0.09)
1–24	85.0	**0.027**	0.3 (0.08)	−0.0 (0.08)
25–48	72.0	0.069	0.2 (0.07)	0.2 (0.10)

^a^Positive numbers indicate first or last activity occurred after sunrise or sunset. Negative numbers indicate first or last activity occurred before sunrise or sunset.

During the next 25–48 days post-treatment, there was no significant difference between the first and last hour of activity at either sunrise or sunset between immune-challenged and control birds; however, trends for experimental birds being active earlier than control birds at dawn and control birds being active marginally later than control birds at dusk were observed (table 2; figure 2). There was no difference in the total number of active hours per day between the two treatment groups (both groups were active on average 11.9 h d^−1^; Mann–Whitney *U*: *W* = 52.5, *p* = 0.942).

When only core daytime hours were analysed, the immune-challenged birds were significantly less active than control birds during core hours within the first 24 h post-treatment (table 3). However, there was no difference in activity levels between immune-challenged and control birds in the time periods for 1–48 h, 1–5 days, 1–20 days, 1–24 days, 25–48 days.

**Table 3.** Generalized linear mixed model outputs for the analysis of activity scores in blackbirds during core daytime hours only (see Methods for model details). Output values for treatment displayed for all time periods analysed (e.g. number of hours or days after lipopolysaccharide injection or control treatment was administered), including the follow-up period of 25–48 days post-treatment. Reference level for treatment shown in brackets.

model	treatment (E)		
	effect	s.e.	*p*
1–24 h	−1.147	0.534	**0.032**
1–48 h	−0.454	0.435	0.297
1–5 days^a^	−0.243	0.320	0.446
1–20 days^a^	−0.196	0.201	0.327
1–24 days^a^	−0.200	0.198	0.313
25–48 days	0.012	0.175	0.945

^a^Model residuals significantly non-uniform—see electronic supplementary material, table S3.

### (c) Baseline immune function and activity

The mean activity score for immune-challenged birds (morning-caught only: *n* = 8) was positively correlated with lysis measured before the experimental immune challenge for the first 24 h of activity post-treatment (Kendall rank tau coefficient: tau = 0.619, *p* = 0.044). This effect was still significant when analysing the first 48 h, even after adjusting the significance threshold for multiple testing (Kendall rank tau coefficient: tau = 0.701, *p* = 0.023), but not for the period 1–5 days when the significance threshold was adjusted for multiple testing (Kendall rank tau coefficient: tau = 0.619, *p* = 0.044). There was no significant correlation between activity score during the first 24 h and baseline immune function for the other three immune parameters (bacterial killing capacity, haptoglobin, agglutination) in the immune-challenged birds (electronic supplementary material, table S4). For control birds (*n* = 7), the same analyses revealed no correlation between any parameters of baseline immune function and mean activity score during the first 24 h (electronic supplementary material, table S4).

## 4. Discussion

This study demonstrates that sickness behaviours in free-living birds last much longer than expected, and that experimentally immune-challenged blackbirds show on average a 19% reduction in activity for as many as 20 days, with the strongest reductions by 59% in the first 24 h. Importantly, the pattern of activity, rather than the overall level of activity during active hours, was largely responsible for the observed effect. In other words, immune-challenged birds ceased activity in the evening earlier than control birds, whereas activity levels of the two treatment groups remained similar during core hours (excluding the first 24 h). During the first 20 days of the experiment, the level of activity in control birds declined to match the activity of experimentally immune-challenged birds, rather than immune-challenged birds increasing their activity to achieve this outcome. This pattern is likely driven by the naturally occurring reduction in activity of control birds towards the end of summer, when moult takes place [58]. This is also indicated by the significant effect of ordinal day in the model results. Thus, while control birds show the expected seasonal decline in activity, the immune-challenged birds already showed reduced activity at a time when they should be more active. Furthermore, our data suggest that baseline (pre-challenge) immune function (complement activity) may modulate the intensity of sickness behaviours. Thus, this study provides a novel insight into how free-living animals express sickness behaviour.

The longevity of sickness behaviours exhibited by immune-challenged birds here was unexpected. Whereas previous studies (with a duration of up to 22 days and carried out on species both smaller and larger than blackbirds) have reported that differences in activity levels between control and immune-challenged groups occur between 3 h and approximately 9 days after treatment [8,13,15–17,20,35–37], here immune-challenged birds were less active than controls during the first 20 days post-treatment. Several factors may have contributed to this sickness behaviour being exhibited for a markedly longer time period than previously reported. Firstly, most experiments for sickness behaviours have been conducted in aviary/caged environments that may preclude individuals from displaying the full extent or intensity of behavioural effects. Such a phenomenon has been demonstrated in LPS-challenged chickens, where free-ranging birds always presented more acute sickness behaviours than those that were caged [36]. Additionally, captive studies often provide food ad libitum, which may reduce energetic constraints, thus decreasing the necessity for more conserved activity. Secondly, long-photoperiod days are known to promote stronger sickness behaviours than short-photoperiod days [20]. All birds in this study were monitored in late summer, which may have influenced the intensity or longevity of the response. Therefore, The time of year when the experiment was conducted may coincide with a time when the birds can afford to show sickness behaviours. Finally, there are no studies (to our knowledge) that have collected consistent behavioural data for free-living, immune-challenged individuals over multiple weeks; thus the full time frame relevant for sickness behaviours in an environmental setting has not been previously examined.

Here, we were able to surmise that ‘wakefulness’ (defined here as the timepoint when birds become, or cease being, active each day) was likely to be the main contributor to the differences seen in overall activity between the two treatment groups after the initial 24 h period. Though we have no data on brain activity to ascertain whether birds were sleeping or simply just sitting still, somnolence has been repetitively cited as one of the most common and notable sickness behaviours associated with infections [1,14,23]. This behaviour is not always exhibited by all bird species [23]; however, our data provide evidence that blackbirds do indeed exhibit some form of this sickness behaviour and that it lasts for a significant period of time. Interestingly, wakefulness (activity) at dusk was reduced more in immune-challenged birds than wakefulness at dawn. This can possibly be explained by energetic or metabolic constraints upon waking that force birds to feed [62]. Alternatively, but not mutually exclusively, foraging conditions might be optimal for this species at dawn as long as the vegetation is damp, which encourages earthworms (blackbirds' main prey) to surface. Additionally, some captive studies report that sickness behaviours can be dampened in response to social cues or resource availability [13,20,22]. Although data collection for our study took place during the end of breeding season and start of the moulting season (both resource-demanding periods), it is not possible to confirm whether overall activity levels or diurnal activity patterns signalled any such dampening effect.

In free-living animals, infection-associated alterations to activity (in particular wakefulness over a period of 20 days) could seriously impede multiple aspects of the species ecology if the effect is similar in other annual-cycle stages. For example, this could easily translate into disadvantageous scenarios, such as constrained capacity to feed young, increased vulnerability to crepuscular predation, reduced refuelling capacities in migrating animals, or a reduced ability to gain sufficient energy stores to roost overnight, particularly during winter. Thus, the length of sickness behaviours observed here has the potential to impact crucial life-history decisions, with possible implications for individual fitness. That birds in our study maintained activity levels in the core hours, but sacrificed the number of active hours (rather than maintaining the number of active hours at lower activity levels throughout the day), may be an adaptive strategy. This strategy could allow individuals to capitalize on optimal foraging or social scenarios [63], especially if they are time-limited. In addition, this strategy may be more effective to avoid predation, as lethargic individuals in the open are easy prey compared with those that are fully alert and active [19], or resting in seclusion. However, reducing the number of active hours will not always be possible, for example when crossing large migration barriers, during periods of food scarcity (e.g. over winter), or when feeding young. It may therefore be these scenarios where even non-lethal immune challenges and their associated sickness behaviours impact individual fitness the most.

Our results also suggest that baseline immune function, in the form of complement activity (lysis), may have predisposed some individuals to better cope with the immune challenge than others. This was exhibited by the positive correlation between pre-challenge (baseline) lysis and the first 24 h and first 48 h of activity in immune-challenged birds after treatment. Complement activity plays an important role in the clearance of bacterial pathogens [52] and since lysis was measured using only plasma rather than whole blood, our results may even be conservative. Therefore, we hypothesize that individuals with higher lysis titres would be better equipped to clear LPS endotoxins efficiently owing to higher complement concentration and thus exhibit sickness behaviours less intensely or over a shorter time frame [64]. This may also provide a mechanistic explanation for why some studies found a correlation between lysis and survival [27,30]. However, further data are required to clarify this relationship owing to the small sample size available, and experimental studies are needed to test for causality. Other measures of baseline immune function were not correlated with mean activity of immune-challenged birds, which in some instances was mechanistically surprising, but could be due to the limited between-individual variation. Nevertheless, our data hint at a mechanistic link between constitutive immune function and sickness behaviours during an immune response.

The start of the experimental period varied between birds and between years. While we made every attempt to distribute control and experimentally immune-challenged birds equalling among the sexes and took age variation into account, we could not control which birds survived until the subsequent breeding season and which accelerometers we could retrieve. As a consequence, deployment dates for retrieved accelerometers are not perfectly matched over time. We have tried to account for this unwanted variation by including ordinal day as covariate in our models, which controls for linear effects such as changes in daylight hours and progress of moult. We acknowledge that this cannot control for stochastic events such as weather. However, during late summer, weather conditions with large abiotic differences do not usually occur abruptly in southern Sweden. Hence, we argue that while the slightly unbalanced design of retrieved accelerometers may have introduced unwanted (yet unavoidable) noise, this is unlikely to have introduced any bias. As such our conclusions may even be conservative.

Overall, this study highlights the potential longevity of behavioural changes in free-living animals during a non-lethal infection. The novel use of hourly accelerometer data allowed us to identify the time of day when sickness behaviours were exhibited and pointed to somnolence (or some form thereof) as the behaviour type that contributed to the overall reductions in activity observed. Over a short time period (e.g. 24–48 h), sickness behaviours could be problematic for animals in energetically or temporally constrained situations (e.g. when feeding young or when in poor condition), whereas over longer periods of time (as seen here) any adverse behavioural alterations are likely to incur accumulative fitness costs over multiple days or weeks. Moreover, our data represent a mimicked infection, and as such these effects may potentially be even greater during a real pathogenic challenge. In conclusion, this study reports an expected response to an immune challenge, but over an unexpectedly long time frame, and thereby provide a novel insight as to how sickness behaviour is necessitated and/or regulated during an infection *in situ*.

## Data Availability

All data are archived in Dryad: https://doi.org/10.5061/dryad.05qfttf7g [65]. Supplementary material is available online [66].

## References

[1] Dantzer R. 2004 Cytokine-induced sickness behaviour: a neuroimmune response to activation of innate immunity. Eur. J. Pharmacol. **500**, 399-411. (10.1016/j.ejphar.2004.07.040) 15464048

[2] Hasselquist D, Nilsson JÅ. 2012 Physiological mechanisms mediating costs of immune responses: what can we learn from studies of birds? Anim. Behav. **83**, 1303-1312. (10.1016/j.anbehav.2012.03.025)

[3] Hegemann A, Alcalde Abril P, Sjöberg S, Muheim R, Alerstam T, Nilsson JÅ, Hasselquist D. 2018 A mimicked bacterial infection prolongs stopover duration in songbirds – but more pronounced in short- than long-distance migrants. J. Anim. Ecol. **87**, 1698-1708. (10.1111/1365-2656.12895) 30101481

[4] Romano A, Rubolini D, Ambrosini R, Saino N. 2014 Early exposure to a bacterial endotoxin may cause breeding failure in a migratory bird. Ethol. Ecol. Evol. **26**, 80-85. (10.1080/03949370.2013.800912)

[5] Bonneaud C, Mazuc J, Gonzalez G, Haussy C, Chastel O, Faivre B, Sorci G. 2003 Assessing the cost of mounting an immune response. Am. Nat. **161**, 367-379. (10.1086/346134) 12703483

[6] Serra L et al. 2012 Seasonal decline of offspring quality in the European starling *Sturnus vulgaris*: an immune challenge experiment. Behav. Ecol. Sociobiol. **66**, 697-709. (10.1007/s00265-012-1318-3)

[7] Risely A, Klaassen M, Hoye BJ. 2018 Migratory animals feel the cost of getting sick: a meta-analysis across species. J. Anim. Ecol. **87**, 301-314. (10.1111/1365-2656.12766) 28994103

[8] Owen-Ashley NT, Turner M, Hahn TP, Wingfield JC. 2006 Hormonal, behavioral, and thermoregulatory responses to bacterial lipopolysaccharide in captive and free-living white-crowned sparrows (*Zonotrichia leucophrys gambelii*). Horm. Behav. **49**, 15-29. (10.1016/j.yhbeh.2005.04.009) 15967447

[9] Eraud C, Jacquet A, Faivre B. 2009 Survival cost of an early immune soliciting in nature. Evolution **63**, 1036-1043. (10.1111/j.1558-5646.2008.00540.x) 19055677

[10] Ilmonen P, Taarna T, Hasselquist D. 2000 Experimentally activated immune defence in female pied flycatchers results in reduced breeding success. Proc. R. Soc. Lond. B **267**, 665-670. (10.1098/rspb.2000.1053) PMC1690582 10821610

[11] Ots I, Kerimov AB, Ivankina EV, Ilyina TA, Horak P. 2001 Immune challenge affects basal metabolic activity in wintering great tits. Proc. R. Soc. Lond. B **268**, 1175-1181. (10.1098/rspb.2001.1636) PMC1088724 11375106

[12] Hart B. 2010 Beyond fever: comparative perspectives on sickness behavior. Encycl. Anim. Behav. **1**, 205-210. (10.1016/B978-0-08-045337-8.00133-9)

[13] Lopes PC, Adelman J, Wingfield JC, Bentley GE. 2012 Social context modulates sickness behavior. Behav. Ecol. Sociobiol. **66**, 1421-1428. (10.1007/s00265-012-1397-1)

[14] Hart BL. 1988 Biological basis of the behavior of sick animals. Neurosci. Biobehav. Rev. **12**, 123-137. (10.1016/S0149-7634(88)80004-6) 3050629

[15] Love AC, Foltz SL, Adelman JS, Moore IT, Hawley DM. 2016 Changes in corticosterone concentrations and behavior during *Mycoplasma gallisepticum* infection in house finches (*Haemorhous mexicanus*). Gen. Comp. Endocrinol. **235**, 70-77. (10.1016/j.ygcen.2016.06.008) 27288634

[16] Sköld-Chiriac S, Nord A, Nilsson J-Å, Hasselquist D. 2014 Physiological and behavioral responses to an acute-phase response in zebra finches: immediate and short-term effects. Physiol. Biochem. Zool. **87**, 288-298. (10.1086/674789) 24642546

[17] Johnson R, Curtis S, Dantzer R, Bahr J, Kelley K. 1993 Sickness behavior in birds caused by peripheral or central injection of endotoxin. Physiol. Behav. **53**, 343-348. (10.1016/0031-9384(93)90215-2) 8446696

[18] Ghai RR, Fugere V, Chapman CA, Goldberg TL, Davies TJ. 2015 Sickness behaviour associated with non-lethal infections in wild primates. Proc. R. Soc. B **282**, 20151436. (10.1098/rspb.2015.1436) PMC4571704 26311670

[19] Adelman JS, Mayer C, Hawley DM. 2017 Infection reduces anti-predator behaviors in house finches. J. Avian Biol. **48**, 519-528. (10.1111/jav.01058) 29242677 PMC5724792

[20] Owen-Ashley NT, Hasselquist D, Raberg L, Wingfield JC. 2008 Latitudinal variation of immune defense and sickness behavior in the white-crowned sparrow (*Zonotrichia leucophrys*). Brain Behav. Immun. **22**, 614-625. (10.1016/j.bbi.2007.12.005) 18255257

[21] Nord A, Hegemann A, Folkow LP. 2020 Reduced immune responsiveness contributes to winter energy conservation in an Arctic bird. J. Exp. Biol. **223**, jeb.219287. (10.1242/jeb.219287) 32341183

[22] Wilsterman K, Alonge MM, Ernst DK, Limber C, Treidel LA, Bentley GE. 2020 Flexibility in an emergency life-history stage: acute food deprivation prevents sickness behaviour but not the immune response. Proc. R. Soc. B **287**, 20200842. (10.1098/rspb.2020.0842) PMC7329051 32546100

[23] Lopes PC, French SS, Woodhams DC, Binning SA. 2021 Sickness behaviors across vertebrate taxa: proximate and ultimate mechanisms. J. Exp. Biol. **224**, jeb225847. (10.1242/jeb.225847) 33942101

[24] Wilcoxen TE, Boughton RK, Schoech SJ. 2010 Selection on innate immunity and body condition in Florida scrub-jays throughout an epidemic. Biol. Lett. **6**, 552-554. (10.1098/rsbl.2009.1078) 20164081 PMC2936216

[25] Matson KD, Horrocks NPC, Versteegh MA, Tieleman BI. 2012 Baseline haptoglobin concentrations are repeatable and predictive of certain aspects of a subsequent experimentally-induced inflammatory response. Comp. Biochem. Physiol. A **162**, 7-15. (10.1016/j.cbpa.2012.01.010) 22316629

[26] Vermeulen A, Eens M, Zaid E, Muller W. 2016 Baseline innate immunity does not affect the response to an immune challenge in female great tits (*Parus major*). Behav. Ecol. Sociobiol. **70**, 585-592. (10.1007/s00265-016-2077-3)

[27] Hegemann A, Marra PP, Tieleman BI. 2015 Causes and consequences of partial migration in a passerine bird. Am. Nat. **186**, 531-546. (10.1086/682667) 26655576

[28] Hanssen S, Hasselquist D, Folstad I, Erikstad K. 2004 Costs of immunity: immune responsiveness reduces survival in a vertebrate. Proc. R. Soc. Lond. B **271**, 925-930. (10.1098/rspb.2004.2678) PMC1691677 15255047

[29] Tella JL, Bortolotti GR, Dawson RD, Forero MG. 2000 The T-cell-mediated immune response and return rate of fledgling American kestrels are positively correlated with parental clutch size. Proc. R. Soc. Lond. B **267**, 891-895. (10.1098/rspb.2000.1086) PMC1690616 10853731

[30] Roast MJ, Aranzamendi NH, Fan M, Teunissen N, Hall MD, Peters A. 2020 Fitness outcomes in relation to individual variation in constitutive innate immune function. Proc. R. Soc. B **287**, 20201997. (10.1098/rspb.2020.1997) PMC7735263 33143586

[31] Legagneux P et al. 2014 No selection on immunological markers in response to a highly virulent pathogen in an Arctic breeding bird. Evol. Appl. **7**, 765-773. (10.1111/eva.12180) 25469158 PMC4227857

[32] Owen-Ashley NT, Wingfield JC. 2007 Acute phase responses in passerine birds: characterization and seasonal variation. J. Ornithol. **148**, 583-591. (10.1007/s10336-007-0197-2)

[33] Bilbo SD, Drazen DL, Quan N, He L, Nelson RJ. 2002 Short day lengths attenuate the symptoms of infection in Siberian hamsters. Proc. R. Soc. Lond. B **269**, 447-454. (10.1098/rspb.2001.1915) PMC1690914 11886635

[34] Mallon EB, Brockmann A, Schmid-Hempel P. 2003 Immune response inhibits associative learning in insects. Proc. R. Soc. Lond. B **270**, 2471-2473. (10.1098/rspb.2003.2456) PMC1691534 14667337

[35] Schultz EM, Hahn TP, Klasing KC. 2017 Photoperiod but not food restriction modulates innate immunity in an opportunistic breeder, *Loxia curvirostra*. J. Exp. Biol. **220**, 722-730. (10.1242/jeb.149898) 27956484

[36] Gregory N, Payne S, Devine C, Cook C. 2009 Effect of lipopolysaccharide on sickness behaviour in hens kept in cage and free range environments. Res. Vet. Sci. **87**, 167-170. (10.1016/j.rvsc.2009.01.003) 19201433

[37] Sköld-Chiriac S, Nord A, Tobler M, Nilsson J-Å, Hasselquist D. 2015 Body temperature changes during simulated bacterial infection in a songbird: fever at night and hypothermia during the day. J. Exp. Biol. **218**, 2961-2969. (10.1242/jeb.122150) 26232416

[38] Adelman JS, Cordoba-Cordoba S, Spoelstra K, Wikelski M, Hau M. 2010 Radiotelemetry reveals variation in fever and sickness behaviours with latitude in a free-living passerine. Funct. Ecol. **24**, 813-823. (10.1111/j.1365-2435.2010.01702.x)

[39] van Dijk JGB, Kleyheeg E, Soons MB, Nolet BA, Fouchier RAM, Klaassen M. 2015 Weak negative associations between avian influenza virus infection and movement behaviour in a key host species, the mallard *Anas platyrhynchos*. Oikos **124**, 1293-1303. (10.1111/oik.01836)

[40] Latorre-Margalef N et al. 2009 Effects of influenza A virus infection on migrating mallard ducks. Proc. R. Soc. B **276**, 1029-1036. (10.1098/rspb.2008.1501) PMC2679067 19129127

[41] van Gils JA, Munster VJ, Radersma R, Liefhebber D, Fouchier RAM, Klaassen M. 2007 Hampered foraging and migratory performance in swans infected with low-pathogenic avian influenza A virus. PLoS ONE **2**, e184-e186. (10.1371/journal.pone.0000184) 17264886 PMC1773019

[42] Svensson L. 1992 Identification guide to European passerines, 4th edn. Stockholm, Sweden: British Trust for Ornithology.

[43] Rappole JH, Tipton AR. 1991 New harness design for attachment of radio transmitters to small passerines. J. Field Ornithol. **62**, 335-337.

[44] Bäckman J, Anderson A, Alerstam T, Pedersen L, Sjöberg S, Thorup K, Tottrup AP. 2017 Activity and migratory flights of individual free-flying songbirds throughout the annual cycle: method and first case study. J. Avian Biol. **48**, 309-319. (10.1111/jav.01068)

[45] Grindstaff JL, Hasselquist D, Nilsson JA, Sandell M, Smith HG, Stjernman M. 2006 Transgenerational priming of immunity: maternal exposure to a bacterial antigen enhances offspring humoral immunity. Proc. R. Soc. B **273**, 2551-2557. (10.1098/rspb.2006.3608) PMC1634911 16959648

[46] Martin LB, Coon CA, Liebl AL, Schrey AW. 2014 Surveillance for microbes and range expansion in house sparrows. Proc. R. Soc. B **281**, 20132690. (10.1098/rspb.2013.2690) PMC3843847 24258722

[47] Hegemann A, Matson KD, Versteegh MA, Tieleman BI. 2012 Wild skylarks seasonally modulate energy budgets but maintain energetically costly inflammatory immune responses throughout the annual cycle. PLoS ONE **7**, e36358. (10.1371/journal.pone.0036358) 22570706 PMC3343055

[48] French SS, Neuman-Lee LA. 2012 Improved *ex vivo* method for microbiocidal activity across vertebrate species. Biol. Open **1**, 482-487. (10.1242/bio.2012919) 23213440 PMC3507210

[49] Eikenaar C, Hegemann A. 2016 Migratory common blackbirds have lower innate immune function during autumn migration than resident conspecifics. Biol. Lett. **12**, 20160078. (10.1098/rsbl.2016.0078) 27029839 PMC4843231

[50] Liebl AL, Martin II LB. 2009 Simple quantification of blood and plasma antimicrobial capacity using spectrophotometry. Funct. Ecol. **23**, 1091-1096. (10.1111/j.1365-2435.2009.01592.x)

[51] Quaye IK. 2008 Haptoglobin, inflammation and disease. Trans. R. Soc. Trop. Med. Hyg. **102**, 735-742. (10.1016/j.trstmh.2008.04.010) 18486167

[52] Matson KD, Ricklefs RE, Klasing KC. 2005 A hemolysis-hemagglutination assay for characterizing constitutive innate humoral immunity in wild and domestic birds. Dev. Comp. Immunol. **29**, 275-286. (10.1016/j.dci.2004.07.006) 15572075

[53] Hegemann A, Pardal S, Matson KD. 2017 Indices of immune function used by ecologists are mostly unaffected by repeated freeze-thaw cycles and methodological deviations. Front. Zool. **14**, 43. (10.1186/s12983-017-0226-9) 28883887 PMC5580329

[54] R Development Core Team. 2020 *R, a language and environment for statistical computing.* Vienna, Austria: R Foundation for Statistical Computing. See https://www.R-project.org/.

[55] Brooks ME, Kristensen K, van Benthem KJ, Magnusson A, Berg CW, Nielsen A, Skaug HJ, Maechler M, Bolker BM. 2017 glmmTMB balances speed and flexibility among packages for zero-inflated generalized linear mixed modeling. R J. **9**, 378-400. (10.32614/RJ-2017-066)

[56] Hartig F. 2020. *DHARMa: residual diagnostics for hierarchical (multi-level/mixed) regression models. R package version 0.3, 3(5).* See https://cran.r-project.org/web/packages/DHARMa/index.html.

[57] Thieurmel B, Elmarhraoui A, Thieurmel MB. 2019 Package ‘suncalc’. See https://cran.r-project.org/web/packages/suncalc/suncalc.pdf.

[58] Jenni L, Winkler R. 2020 The biology of moult in birds. London, UK: Bloomsbury Publishing.

[59] Morales A, Frei B, Mitchell GW, Bégin-Marchand C, Elliott KH. 2022 Reduced diurnal activity and increased stopover duration by molting Swainson's thrushes. Ornithology **139**, ukab083. (10.1093/ornithology/ukab083)

[60] Lüdecke D. 2018 ggeffects: Tidy data frames of marginal effects from regression models. J. Open Source Softw. **3**, 772. (10.21105/joss.00772)

[61] Zylberberg M. 2015 Common measures of immune function vary with time of day and sampling protocol in five passerine species. J. Exp. Biol. **218**, 757-766. (10.1242/jeb.111716) 25617452

[62] Bonter DN, Zuckerberg B, Sedgwick CW, Hochachka WM. 2013 Daily foraging patterns in free-living birds: exploring the predation–starvation trade-off. Proc. R. Soc. B **280**, 20123087. (10.1098/rspb.2012.3087) PMC3652453 23595267

[63] McNamara J, Mace R, Houston A. 1987 Optimal daily routines of singing and foraging in a bird singing to attract a mate. Behav. Ecol. Sociobiol. **20**, 399-405. (10.1007/BF00302982)

[64] Reid RR, Prodeus AP, Khan W, Hsu T, Rosen FS, Carroll MC. 1997 Endotoxin shock in antibody-deficient mice: unraveling the role of natural antibody and complement in the clearance of lipopolysaccharide. J. Immunol. **159**, 970-975. (10.4049/jimmunol.159.2.970) 9218618

[65] Hegemann A, Lennon R, Ronanki S. 2023 Data from: Immune challenge reduces daily activity period in free-living birds for three weeks. Dryad Digital Repository. (10.5061/dryad.05qfttf7g) PMC10427819 37583320

[66] Lennon RJ, Ronanki S, Hegemann A. 2023 Immune challenge reduces daily activity period in free-living birds for three weeks. Figshare. (10.6084/m9.figshare.c.6771524) PMC10427819 37583320

